# Functional and Structural Diseases

**Published:** 1884-06

**Authors:** 


### FUNCTIONAL AND STRUCTURAL DISEASES.

Reynolds, in the introduction to his "System of Medicine," under the heading, "Structural and Functional Diseases," after saying that these two phases have passed into common usage, adds, further: "Recently it has become somewhat the fashion to object to the latter and to deny the existence of any such condition." At a recent meeting of the Medical Club a discussion occurred upon this question, in which a minority of the members, including the writer, argued the affirmative. At that time the article referred to above had not been read by the writer, the opinion being based upon individual reasoning in general, and especially upon thoughts suggested by a late study of the introductory chapters of Foster's Physiology. From this work we quote the following:

"Among the simpler organisms known to biologists, perhaps the most simple as well as the most common is that which has received the name of amœba. There are many varieties of amœba, and probably many of the forms which have been described are, in reality, merely amœbiform phases in the lives of certain animals or plants, but they all possess the same general characters. Closely resembling the white corpuscles of vertebrate blood, they are wholly or almost wholly composed of undifferentiated protoplasm, in the midst of which lies a nucleus, though this is sometimes absent. In many a distinction may be observed between a more solid external layer or *ectosarc*, and a more fluid granular interior or *endosarc*, but in others even this primary differentiation is wanting. By means of a continually occurring flux of its protoplasmic substance, the amœba is enabled, from moment to moment, not only to change its form but also to shift its position. By flowing round the substances which it meets, it, in a way, swallows them, and having digested and absorbed such parts as are suitable for food, ejects or rather flows away from the useless remnants. It thus lives, moves, eats, grows, and after a time dies, having been, during its whole life, hardly anything more than a minute lump of protoplasm. Hence, to the physiologist it is of the greatest interest, since in its life the problems of physiology are reduced to their simplest forms.

"Now, the study of an amœba, with the help of knowledge gained by the examination of more complex bodies, enables us to state that the undifferentiated protoplasm of which its body is so largely composed possesses certain fundamental vital properties.

"It is irritable and automatic When any disturbance, such as contact with a foreign body, is brought to bear on the amœba at rest, movements result. These are not passive movements, the effects of the push or pull of the disturbing body, proportionate to the force employed to cause them, but active manifestations of the contractility of the protoplasm; that is to say, the disturbing cause or 'stimulus' sets free a certain amount of energy previously latent in the protoplasm, and the energy set free takes on the form of movement. Any living matter which, when acted on by a stimulus, thus suffers an explosion of energy, is said to be 'irritable.' The irritability may, as in the amœba, lead to movement; but in some cases no movement follows the application of the stimulus to irritable matter, the energy set free by the explosion taking on some other form than movement, *ex. gr.*, heat. Thus a substance may be irritable and yet not contractile, though contractility is a very common manifestation of irritability.

"The amœba (except in its prolonged quiescent stage) is rarely at rest. It is almost continually in motion. The movements cannot always be referred to changes in surrounding circumstances acting as stimuli; in many cases the energy is set free in consequence of internal changes, and the movements which result are called spontaneous or automatic movements. We may, therefore, speak of the protoplasm of the amœba as being irritable and automatic.

"The energy expended in the movements of the amœba is supplied by the chemical changes going on in the protoplasm, by the breaking up of bodies possessing much latent energy into bodies possessing less. Thus the metabolic changes which the food (as distinguished from the undigested stuff mechanically lodged for awhile in the body) undergoes in passing through the protoplasm of the amœba are of three classes: Those preparatory to and culminating in the conversion of the food into protoplasm, those concerned in the discharge of energy, and those tending to economize the immediate products of the second class of changes by rendering them more or less useful in carrying out the first."

In a note, the author explains his meaning as follows:

"This word automatic has recently acquired a meaning almost exactly opposite to that which it originally bore, and an automatic action is now, by many, understood to mean nothing more than an action produced by some machinery or other. In this work I use it in the older sense, as denoting an action of a body, the causes of which appear to lie in the body itself. It seems preferable to 'spontaneous,' inasmuch as it does not necessarily carry with it the idea of irregularity, and bears no reference to a 'will.'"

One of the prominent theories regarding the nature of epilepsy is that the latent energy above referred to is set free and perhaps developed, with abnormal energy and frequency. If an amœba, composed of structureless protoplasm can set free the latent energy stored up in its substance, the difference in its susceptibility to external irritation must be one of function, for structural changes surely cannot occur where structure is absent. This was the line of argument maintained at the meeting referred to. It will be noticed that supposed changes in molecular arrangement (a pure working hypothesis, as yet) are not admitted as change in structure. Upon this point the views of Reynolds are right to the point, and we conclude this article with quotations from this clear thinking author. The reasons which have led him to retain the term "functional diseases," in his "System of Medicine," he gives as follows:

"For this purpose three classes of facts have to be remembered: 1st. That there are some structural alterations, such, for example, as atheroma in the vessels, which may, if an individual has been killed by an accident, be found extensively distributed throughout the body, the existence of which had been neither known nor suspected by the presentation of any functional change, or symptom, during life. On the other hand, a man may have suffered for many years from discomfort, or marked derangement of the functions of the brain, heart, or lungs and yet the most practiced anatomist, with all means and appliances to help him, may fail to discover, post mortem, any organic change which is sufficient to have accounted for them. 2d. Another class of facts, constantly lost sight of by those who deny the existence of functional disease, is to be found in the relations between structure and function in health. At the end of a day's work, and after a night's repose, we might find the two extreme conditions of the organism as regards function. For twelve hours every muscle, nerve and organ has been doing its utmost, and, as we know, has been wearing out. During the hours of sleep, many organs have been doing little, and some nothing, whereas others have, as it seems, to work on without repose; but in all, repair has been going on. By an examination of the body, killed suddenly at the end of one or the other of these periods, it might be possible to infer which had been the condition immediately preceding death. But this inference would be based upon the relation exhibited between the products of functional activity, such as the nature, quantity and quality of the secretions in their several receptacles; and the raw materials upon which the organs have to work, such as the nature, quantity and quality of the chyle, lymph and blood. It would not be formed upon regard directed only to the condition of organs which had been either in activity or repose. It could not be so based, because the process of repair in the living, healthy body is one that is simultaneous and commensurate with waste. The muscles are not mended up as we mend a damaged wall, by patching up a hole here, and binding on an iron brace or girder yonder; but the process is interstitial; new material is brought in, and brought everywhere; the existing organ is worn down, and the waste matter is carried away; but, with all this change, there is a persistent textural result. Looking at this question still more closely, we see that function is related to structure, not only in the sense that it is what the organ does, but in the much more important meaning that it is at once the expression of the wear and also of the repair of tissues; or, in other words, the outcome of their life. In the present state of physiology, it is impossible to conceive of a living organ without believing in the nutritive, molecular changes it is undergoing, and these are the essential conditions of its functional activity; it is equally impossible to imagine the function of any living tissue being called into exercise without recognizing the dependence of this functional operation upon interstitial movements of repair and waste. But we should be wrong, on the other side, were we to confound function with the nutritive changes which constitute, not the function itself, but the conditions of its exercise. It is, for example, the peculiar function of a muscle to shorten itself, of a nerve to convey an impulse either of motion or sensation, and of a nerve-center to convert one of these impulses into the other; the organs referred to, in exercising these functions, undergo certain nutrition-changes; but these molecular changes are not the functions of the organs, but the conditions essential for their performance. This principle, which it seems almost unnecessary to state, in regard of the particular organs or tissues now referred to, is, however, not unfrequently lost sight of in respect of secreting organs. It is the function of the salivary gland to secrete a fluid having special characters; of the liver to do this, and to effect changes in the blood which comes to it; and so of other secreting organs; they receive blood into them, and from all of them it passes away, changed, and the organ, as part product of this change, gives up its secreted matter. These functions, be it observed, depend for their performance upon nutrition-changes in the cells and tissues of the organs; but those fine processes of change are the conditions of functional activity, and are not to be confounded with the thing itself. The secreting cell has to live, to waste, and be repaired; and it lives at a degree of pressure, and is wasted and repaired at a rate, directly proportioned to the amount of work that it accomplishes, and thus it is conditioned precisely as are the ultimate elements of the muscle or the nerve. But minute as is our knowledge of much that goes on in the secreting organs, and of the chemical nature of the results or products of their work, we know no more of the physical conditions which determine that one set of cells shall separate urea, another set saliva, and a third bile acids from the blood, than we do of those which enable one nerve-fibre to convey impressions of light, another of sound, and another those of motion. These are, at present, ultimate facts of physiological science; the function is the expression of the life of the structure; it is what the latter was constructed for the purpose of doing; in doing it, the structure undergoes change; it is wasted and repaired, but these processes are carried on without any breach in the integrity of tissue. Function is to nutrition as electricity is to the chemic changes in the galvanic battery, a "correlated force." We do not say that the one is the other, but that it is converted into it, and, as in the inorganic world, the arrangement and nature of the particular materials with which different forces come into contact, determine whether chemic action shall appear as heat or magnetism, whether heat shall be shown in motion, light, or electricity, so do the different materials of the living organs, and their arrangement, determine the nature of the functions they perform; how they do this, we do not know, but the facts of physiological science are well known, viz., that the nerve-cell exhibits one class of powers, the muscular cell another class, and the secreting organs a third. 3d. A third class of facts to be remembered is, that in many diseases the only symptoms to be recognized are changes in the degree of activity with which certain organs perform their functions. No new element is introduced by some diseases into the category of vital actions. Such affections as chorea, hysteria, epilepsy, might be shown to consist of mere modifications in the degree, time of occurrence, and combinations of functions, each of which, taken *per se*, is consistent with health. The sudden loss of consciousness in epilepsy, for example, is not more mysterious than is the sudden but every-day recurring passage from wakefulness to sleep; the arrested respiration is similar in kind to that seen when the chest is fixed in the performance of any great muscular exertion involving the upper limbs; and still more similar to that which can scarcely be called morbid, the prolonged apnœa of a screaming child, whether the scream be the expression of terror, temper, or pain; the convulsive movements are neither more nor less than nerve and muscular functions, any of which might separately, and many of which might in combination, be the expression of healthy vital activity.

"From these three classes of facts, therefore, we are compelled to admit that, in the present state of science, the *onus probandi* lies with those who assert the constant presence of structural in association with functional change; and we affirm that those who make the assertion have never proved their point. Further, that as a matter of inference from what we know of the relation subsisting between structure and function in health, we should not even expect to find solutions of continuity or coarse changes of texture in those diseases, the essential elements of which are functions altered, not in kind, but only in degree and mode of association; and that, on the other hand, when we do find material changes in association with functional disturbances, we should refer many of the latter only indirectly to what we see of the former, the more numerous and more important of them being dependent upon what we do not see, viz., the finer changes in the interstitial processes of nutrition. A scirrhous tumor of the stomach, for example, may produce certain symptoms easily explicable by its mechanical effects; it may be so situated as to prevent the ingress or egress of food; but vomiting may occur when the orifices are free, or when the tumor is situated in some organ in the pelvis; the supposed tumor may cut off the supply of food, and so explain some of the changes we see in color and general nutrition; but, on the other hand, the extreme of wasting and of cancerous tinting may be seen when there is no such enforced abstinence from food, but when indeed a large quantity is not only taken, but is digested and enjoyed. So again, a clot, of blood in the corpus striatum may sever the nerve-fibres, and so explain the servance between the will and certain muscles of the extremities; but it will not so explain the presence of convulsions, or of spasm in those palsied limbs.

"For these reasons we retain the words 'functional disease,' understanding by them such changes as have no recognized morbid anatomy, but such as depend upon corresponding changes in the finer processes of nutrition. We do not believe that there is any altered function without a correlated change in the nutrition of the organ; but what we assert is that such a change, as a matter of fact, is of such kind as to be undiscoverable by our senses, and as a matter of inference, from what we know of the relation between nutrition and function, is of such nature that it may always be beyond the reach of observation. No healthy function is performed without nutrition-change; no morbid function can exist without altered nutrition-change; but the relation between the two elements, 'structure' and 'function,' is the same in the two conditions.

"Nothing is more erroneous than the common notion that 'functional' means trivial, and that 'structural' means severe. Many diseases, designated by the former word, are long-continued, obstinate, or destructive; many known by the latter are of short duration, are amenable to treatment, or are harmless. Diseases which spoil or shorten life are not trivial because they depend upon such fine changes as may escape our observation; but they are the more serious when they thus elude our notice, just because they have their place in the very centre—the most ultimate process and fact—of life, the conduct of nutrition."

The views above expressed are exactly our own, and we invite criticism of them from those who may have dissenting opinions to offer.

